# Role of exosomal miRNAs in brain metastasis affected by radiotherapy

**DOI:** 10.1515/tnsci-2020-0163

**Published:** 2021-03-31

**Authors:** Zihuang Li, Hongli Yang, Ling Ye, Rencui Quan, Meili Chen

**Affiliations:** Department of Radiation Oncology, The Second Clinical Medical College of Jinan University, Shenzhen Municipal People’s Hospital, 1017 Dongmen North Road, Shenzhen, Guangdong, 518020, China; Department of oncology, The First Affiliated Hospital of Ji Nan University, Guangzhou, Guangdong, China

**Keywords:** radiotherapy, brain metastasis, exosome, microRNAs, malignant tumors

## Abstract

In oncogenesis and development of malignant tumor, microRNAs (miRNAs) regulate the complex gene expression associated with the tumor pathogenesis. Currently, only few studies have been conducted to identify miRNAs and the potential pathways involved in the pathogenesis of brain metastasis in patients who underwent radiotherapy, especially miRNAs in the plasma exosomes. Therefore, this study is aimed to use small RNA analysis to identify miRNAs and their potential target genes in plasma exosomes during the initiation and development of brain metastasis in patients who underwent radiotherapy. Using high-throughput sequencing technologies, we identified 35 differentially expressed miRNAs in patients with brain metastasis who had undergone radiotherapy. In annotation of miRNA targets, gene ontology enrichment analysis revealed that the targets of the differentially expressed miRNAs were significantly enriched in the regulation of cellular processes. Kyoto Encyclopedia of Genes and Genomes revealed that most of the miRNA targets were cancer-related, including genes involved in the mitogen-activated protein kinase signaling pathway, cancer-related pathways, phosphatidylinositol 3-kinase-protein kinase B signaling pathway, microtubule-associated protein kinase signaling pathway, Ras signaling pathway, regulation of the actin cytoskeleton, and axon guidance. In conclusion, this study provides a new perspective to understand the possible function of these miRNAs in the pathogenesis of brain metastasis. This was the first time that a pilot study identified plasma exosomal miRNAs in five patients with brain metastasis before and after radiotherapy. This study is the beginning; more specimen and further research are needed to explore the functional role of specific miRNAs and their potential as therapeutic targets for brain metastasis.

## 1 Introduction

Brain metastases are the most common intracranial malignant tumors, and their primary foci typically include lung cancer (20–56%), breast cancer (5–20%), and melanoma (7–16%), as well as colorectal cancer and renal cell carcinoma [1,2]. Brain metastasis occurs because of the proliferation of cells. In addition, it can occur because of the transport of the primary tumors to cerebral vessels through the blood. Complex niche tumor interactions in the microenvironment, neuroinflammatory cascade, and possible neovascularization are involved in new metastasis [3]. Once brain metastasis occurred in patients with cancer, they induce various central nervous system (CNS) symptoms that severely reduce the quality of life of patients, resulting in poor prognosis and high mortality. If untreated, the survival time is approximately 1–2 months, while the median survival time is only 4–12 months after active treatment [4]. To solve the problem of poor prognosis in patients with brain metastasis, we need to understand the disease complexity at the molecular level. Some hypotheses have been proposed to explain the unique metastasis patterns of different primary cancers, including the importance of “seeds” (cancer cells) and “soil” (microenvironment of receiving organs) or changes in circulation patterns between primary and metastatic tumors. Evidence shows that metastatic cells and tumor microenvironment are essential for tumor growth [3]. It is important to understand the unique biological sensitivity of each tumor and the molecular differences occurring because of the corresponding treatment of brain metastasis. The CNS is protected by various functional barriers, including the blood–brain barrier (BBB) and blood-cerebrospinal fluid (CSF) barrier. Studies have shown that circulating monocytes can interfere with brain homeostasis through the BBB [5,6], and circulating tumor cells may use this mechanism to enter the brain [7]. The blood-CSF barrier is formed by the epithelial cells of the choroid plexus that form tight junctions. The capillary window and intercellular space of the choroid plexus allow molecules to move freely in these compartments [8]. The expression of a complement protein (C3) in primary cancer cells can destroy the blood-CSF barrier, causing mitogens to enter the CSF [9]. These factors may lead to brain metastasis.

Exosomes are heterogeneous nanoscale vesicles found in blood and other body fluids. These disperse a variety of bioactive molecules, such as proteins, mRNA, microRNAs (miRNA), DNA, and lipids, into various cells. Exosomes can cross the BBB, and during inflammatory stimulation, the endothelial cells of the brain release exosomes into the bloodstream by responding to inflammatory reactions. Moreover, inflammatory stimulation leads to the internalization of cells and the formation of early endosomes, which then form exosomes that are finally released into the blood or brain parenchyma [10]. Exosomes, as a mode of communication between the cells and the environment, have increasingly become a focus of research [11,12,13]. They carry thousands of proteins and nucleic acids and transmit biological information over a long distance. Exosomes play an important role in the occurrence and development of brain metastasis. Therapeutic strategies for brain metastasis include radiotherapy, surgery, chemotherapy, immunotherapy, and targeted therapies; in particular, radiotherapy treatments for brain metastasis have developed rapidly [14]. This study is aimed to explore the recognition of differentially expressed miRNAs in plasma exosomes and the prediction of target signaling pathways in patients with brain metastasis before and after radiotherapy treatment, possibly providing new clues for the treatment of brain metastasis.

## 2 Methods

### 2.1 Patient sample collection

Plasma was collected from five patients with lung adenocarcinoma or melanoma diagnosed as brain metastasis (aged 33–75 years) at the Shenzhen People’s Hospital. This study was conducted in accordance with the principles of the Declaration of Helsinki (1964) and with the participants’ understanding and consent. Inclusive criteria: age between 18 and 75 years old; Karnofsky performance status (KPS) score ≥60; number of brain metastasis ≤4; maximum diameter of metastasis ≤3 cm; primary tumor was stable or controlled within 2 months after drug treatment; no other extracranial metastasis; no hard (soft) meningeal metastasis; no history of brain radiotherapy. Exclusion criteria: primary tumor was not controlled; extracranial metastasis occurred simultaneously; history of craniocerebral radiotherapy; pregnant or lactating women; patients not suitable for large fraction irradiation; patients with active liver, kidney, and heart diseases; drug abuse, long-term alcoholism, and AIDS. Withdrawal criteria: unable to carry out treatment according to the requirements of the study protocol; serious adverse events, pregnancy of the patient, and withdrawal of the subject. The clinical specimens included peripheral blood (approximately 4–5 mL), which was collected within 3 days before and after radiotherapy, respectively. The blood was collected in ethylenediaminetetraacetic acid tubes, stored at −4℃, and then centrifuged for 10 min at 12,000 *g*. The plasma was harvested and stored at −80℃ until exosome isolation. All brain metastasis cells were histologically diagnosed using magnetic resonance imaging (MRI). The treatment for patients with brain metastasis was intensity modulated radiation therapy/volumetric modulated arc radiotherapy.


**Ethical approval:** This research related to human use has been complied with all the relevant national regulations and institutional policies and in accordance with the tenets of the Helsinki Declaration, and has been approved by the authors’ institutional review board or equivalent committee.
**Informed consent:** Informed consent has been obtained from all individuals included in this study.

### 2.2 Exosome isolation and RNA extraction

Exosomes were isolated from the plasma using an exoQuick precipitation (System Biosciences, USA) according to the manufacturer’s protocol. Briefly, the exosomes were precipitated by incubation; the exoQuick exosome precipitation reagent was added at 4°C for 60 min. The exosome pellet was collected by centrifugation at 1,500 × *g* for 10 min at 4°C and resuspended in 10 mM PBS to four times the original plasma volume. Nanoparticle tracking analysis and transmission electron microscopy were used to identify the exosomes. Subsequently, total RNA was extracted from exosome pellets using TRIzol (Thermo Fisher Scientific, Inc., Carlsbad, CA, USA), followed by treatment with DNase to remove potential DNA contamination. The integrity and concentration of the RNA were determined photometrically at 260 nm and 280 nm using an Agilent 2100 Bioanalyzer (Agilent Technologies, USA).

### 2.3 Small RNA library construction and sequencing

Approximately 1 µg of total RNA per sample was used for the RNA sample preparations. Small RNA library preparation was conducted using TruSeq Small RNA Sample Prep Kits (Illumina, San Diego, USA). After ensuring sample purity with an Agilent 2100 Bioanalyzer, the purified libraries were sequenced on an Illumina HiSeq 2500.

### 2.4 miRNA-sequencing data analysis

Quality control was performed on raw data using the FASTX-Toolkit, which involved the removal of low-quality reads and trimming adapters. Clean reads were aligned to the human reference transcriptome sequence. Reads per kilobase of exon model per million mapped reads were calculated to obtain normalized expression levels. Finally, differential expression analysis was performed using edgeR. Before differential expression analysis, for each sequenced library, the read counts were adjusted using one scaling normalized factor. The relative counts of miRNAs were compared with the corresponding values before and after radiotherapy. The *p*-value was adjusted using the Benjamini–Hochberg method. The miRNAs with corrected *p*-values <0.05 were identified as significantly differentially expressed miRNAs.

### 2.5 Gene ontology function and Kyoto Encyclopedia of Genes and Genomes pathway enrichment analysis

Potential target genes of differentially expressed miRNAs were predicted using the popular database TargetScan. Gene ontology (GO) function enrichment analysis was performed using the topGO software (version 2.18.0), and Fisher’s exact test was used for statistical analysis. Kyoto Encyclopedia of Genes and Genomes (KEGG) pathways were analyzed using KOBAS software; *p*-value <0.05 indicated a significant difference.

### 2.6 Quantitative reverse transcription-polymerase chain reaction validation of the differentially expressed miRNAs

To validate the miRNA expression results from sequencing, total RNA from the exosomes was extracted using a TaqMan^®^ MicroRNA Reverse Transcription kit (Thermo Fisher Scientific, Inc., Carlsbad, CA, USA) and analyzed by quantitative reverse transcription-polymerase chain reaction (RT-qPCR) as per manufacturer’s instructions. Briefly, qPCR was carried out using SYBR‑Green (Thermo Fisher Scientific, Inc.) according to the manufacturer’s instructions in an ABI 7300 real‑time qPCR system (Thermo Fisher Scientific, Inc., Carlsbad, CA, USA). The PCR conditions were as follows: 95℃ for 3 min, followed by 45 cycles at 95℃ for 10 s, 60℃ for 45 s, and 72℃ for 30 s. The quantification of miRNAs was performed in relation to the U6 housekeeping miRNA. The relative abundance of miRNAs was calculated using the comparative Cq method (2^−ΔΔCq^) and assessed by *t*-test.

## 3 Results

### 3.1 Brain metastasis miRNA signature derived from plasma exosomes

**Figure 1:**
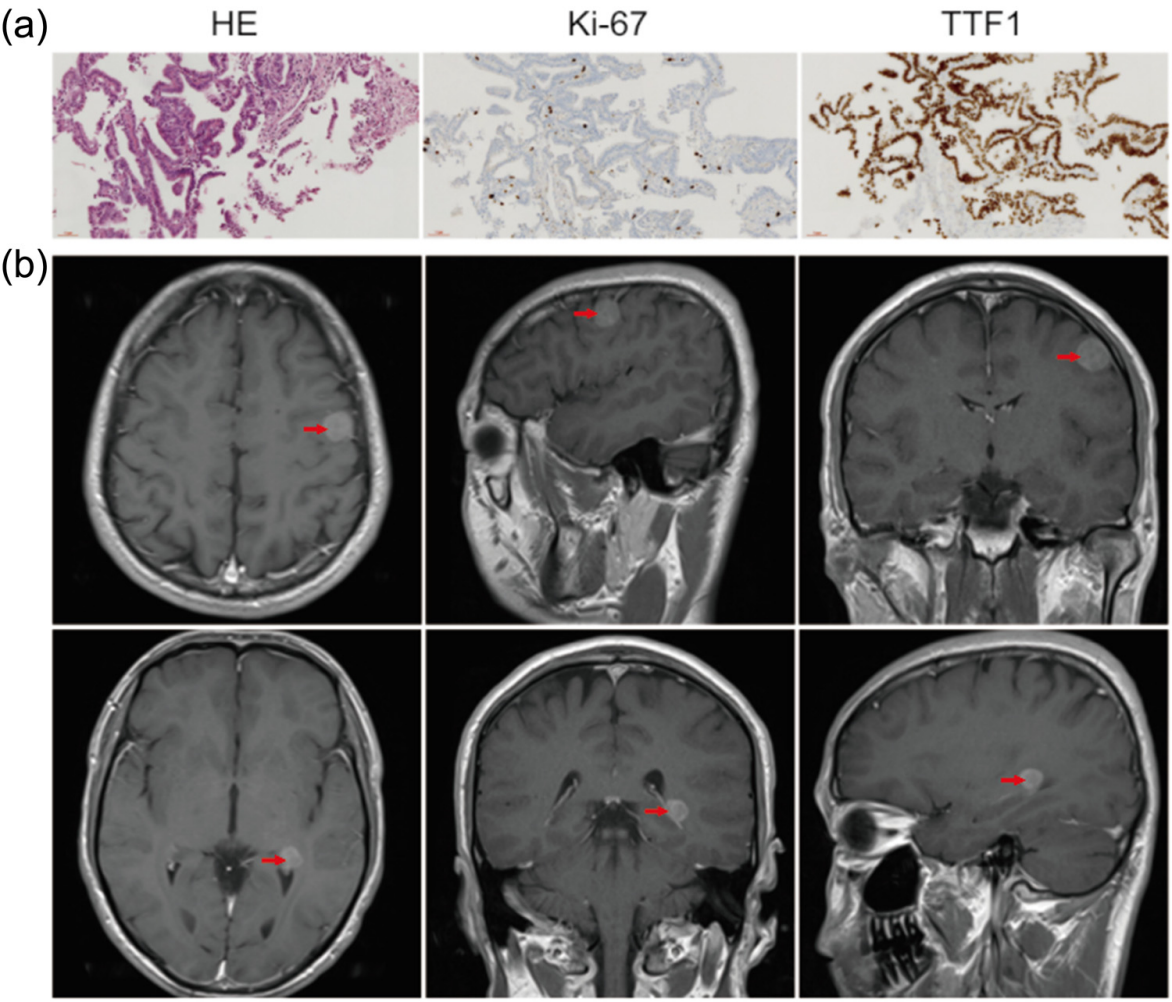
Brain metastases were diagnosed histologically and MRI in lung adenocarcinoma clinical samples. (a) The expression of tumor-related indexes was detected by immunohistochemistry with 20× objective lens in lung adenocarcinoma. (b) Imaging examination of multiple brain metastases in lung adenocarcinoma.

**Figure 2:**
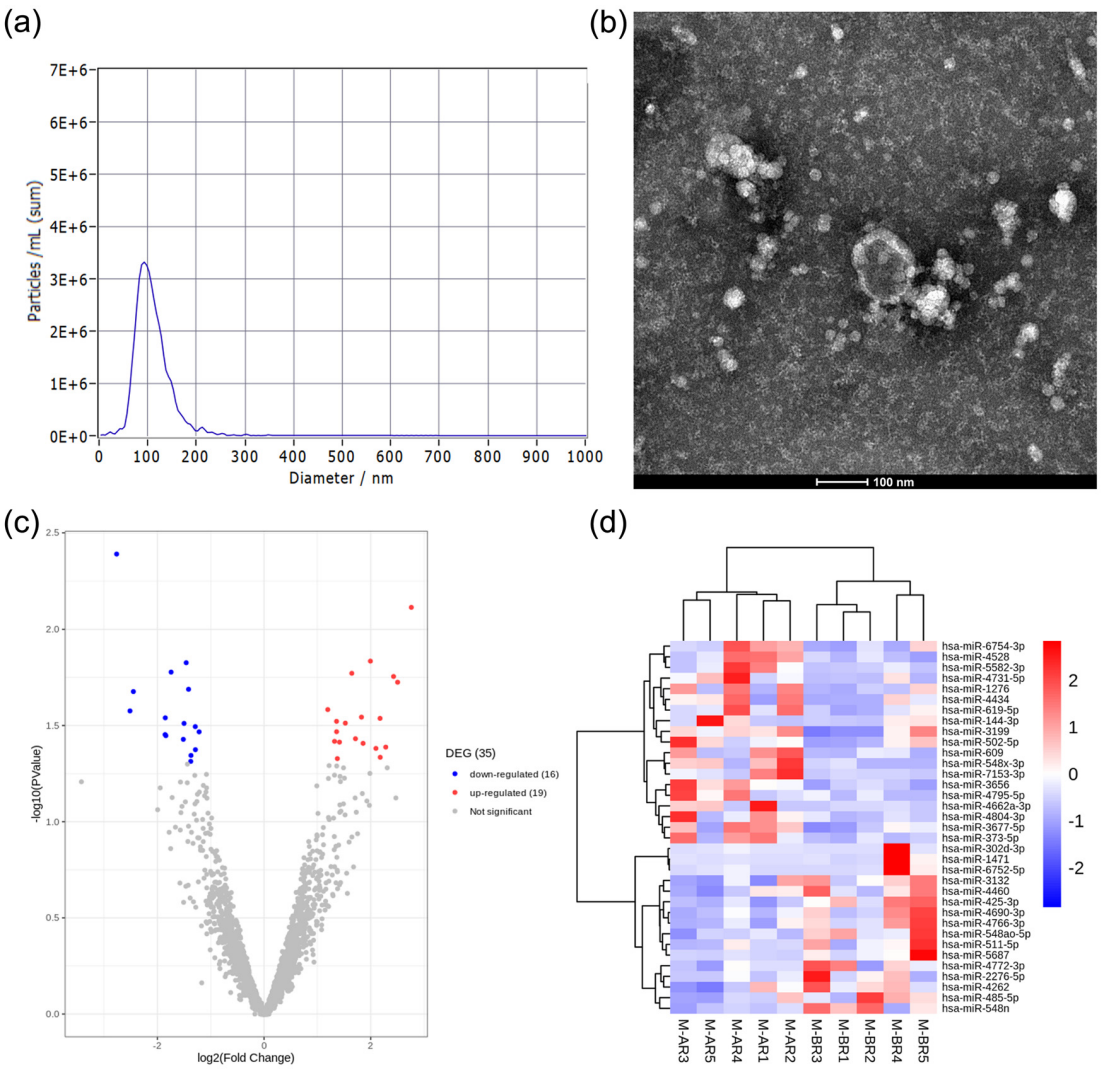
Identification of exosomes and the expression of differentially expressed miRNAs in exosomes. (a and b) Measurement of diameter of vesicles to identify exosomes by nanoparticle tracking analysis and transmission electron microscopy. (c and d) Volcano plot and heatmap showed the differentially expressed miRNAs in each sample. M-AR, metastasis after radiotherapy; M-BR, metastasis before radiotherapy.

For the global analysis of the miRNA signature derived from plasma exosomes of brain metastasis, ten clinical plasma exosomes were collected for sequence analysis, including five brain metastasis samples before radiotherapy (M-BR) and five after radiotherapy (M-AR) (Table 1). The primary lesions and brain metastasis were detected by immunohistochemistry and MRI, respectively, as shown in Figure 1. To identify significantly differentially expressed miRNAs in plasma exosomes from pre-and post-radiotherapy samples, the screening threshold was set to a fold change of >2 and a *p*-value <0.05. A total of 35 differentially expressed miRNAs were identified, including 16 miRNAs that were downregulated following radiotherapy (miR-5687, miR-4766-3p, miR-4690-3p, miR-4262, miR-302d-3p, miR-6752-5p, miR-548ao-5p, miR-4772-3p, miR-485-5p, miR-511-5p, miR-1471, miR-2276-5p, miR-548n, miR-3132, miR-425-3p, and miR-4460) and 19 that were upregulated (miR-7153-3p, miR-609, miR-373-5p, miR-5582-3p, miR-4662a-3p, miR-619-5p, miR-3656, miR-502-5p, miR-6754-3p, miR-4804-3p, miR-3199, miR-4434, miR-3677-5p, miR-4528, miR-4731-5p, miR-144-3p, miR-548x-3p, miR-4795-5p, and miR-1276) (Figure 2).

**Table 1:** Sample pathological diagnosis information (M-BR, metastases before radiotherapy)

Specimens	Age (years)	Diagnosis	Single/multiple	Metastasis sites	Size of brain metastases (mm)
M-BR1	75	Melanoma brain metastases	Single	Left frontal lobe	28 × 16
M-BR2	36	Lung adenocarcinoma brain metastases	Multiple	Left frontal lobe and right temporal pole	25 × 20
M-BR3	33	Lung adenocarcinoma brain metastases	Multiple	Intracranial supratentorial	15 × 15
M-BR4	47	Lung adenocarcinoma brain metastases	Multiple	Left parietal lobe and occipital bone	14 × 11
M-BR5	75	Lung adenocarcinoma brain metastases	Multiple	Bilateral brain parenchyma	28 × 24

### 3.2 GO enrichment analysis

**Figure 3:**
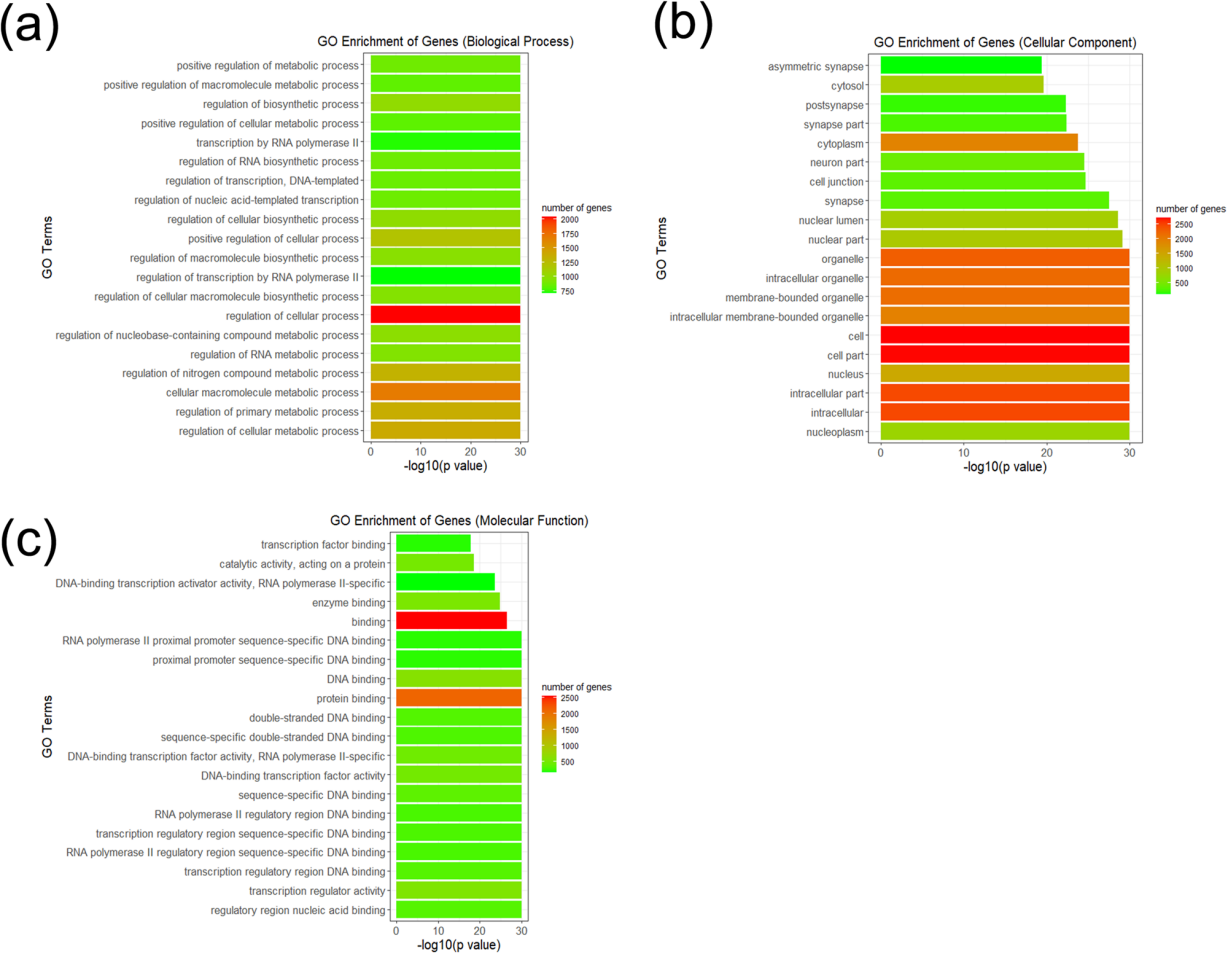
Top 20 functional GO terms of the differentially expressed miRNA target genes shown according to *p*-value. (a–c) Biological process, cellular component, and molecular function terms of the differentially expressed miRNA target genes.

GO enrichment analysis was performed on significantly differentially expressed miRNA target genes predicted by TargetScan, and the related biological processes, cellular components, molecular functions, and gene numbers were analyzed. The most significantly enriched GO terms for the biological process group were regulation of cellular processes and cellular macromolecule metabolic processes. In the cellular component category, many target genes were found in the nucleoplasm as well as intracellular components. In the molecular function category, the areas of regulation included nucleic acid binding, transcription regulator activity, and protein binding (Figure 3). The result of GO enrichment analysis showed that the significantly differentially expressed miRNAs in plasma exosomes were associated with metabolic processes and transcription regulator activity, which were associated with cancer-associated pathways, and may play an important role in brain metastasis.

### 3.3 KEGG pathway analysis

**Figure 4:**
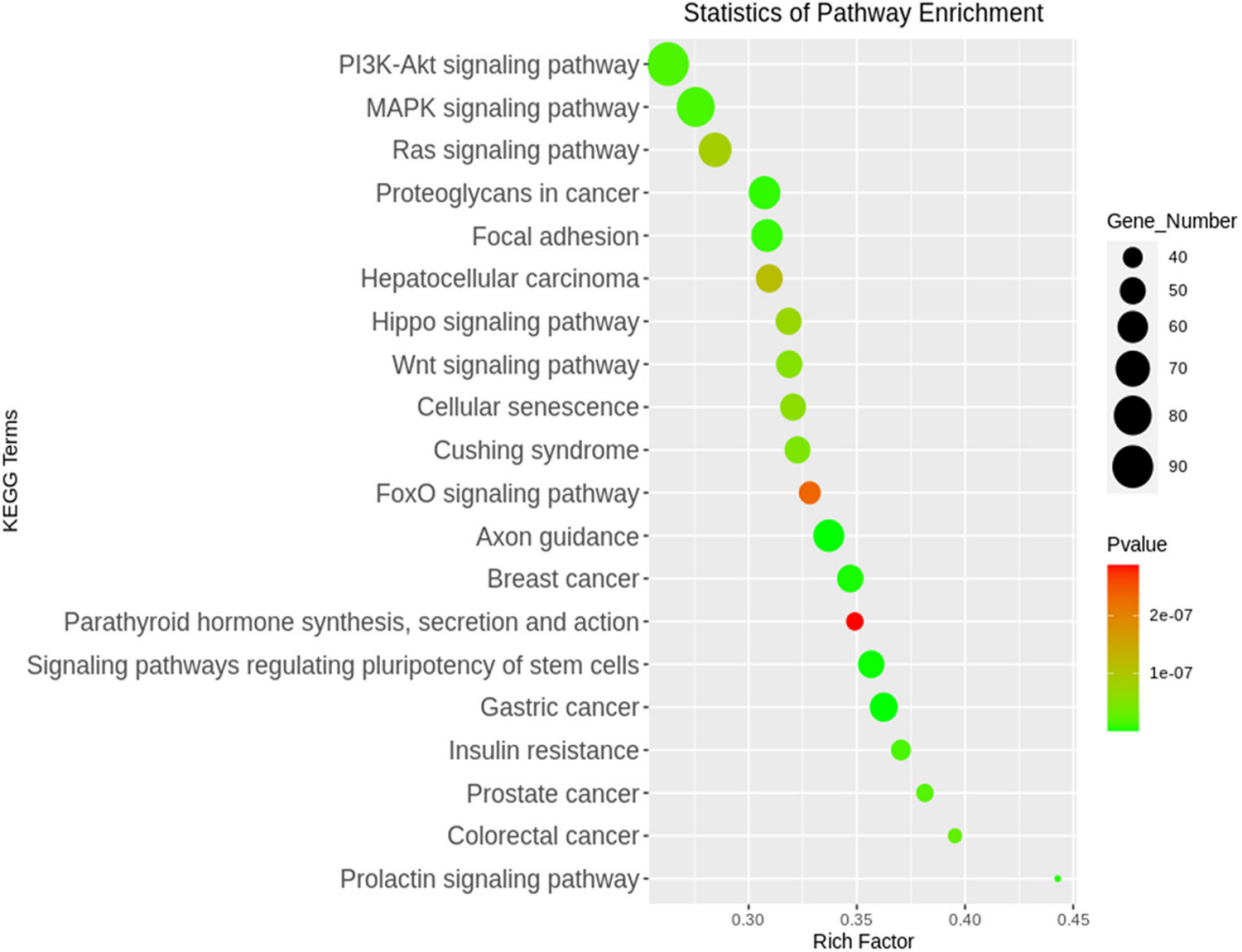
Top 20 KEGG pathways of the differentially expressed miRNA target genes shown according to *p*-value.

**Figure 5:**
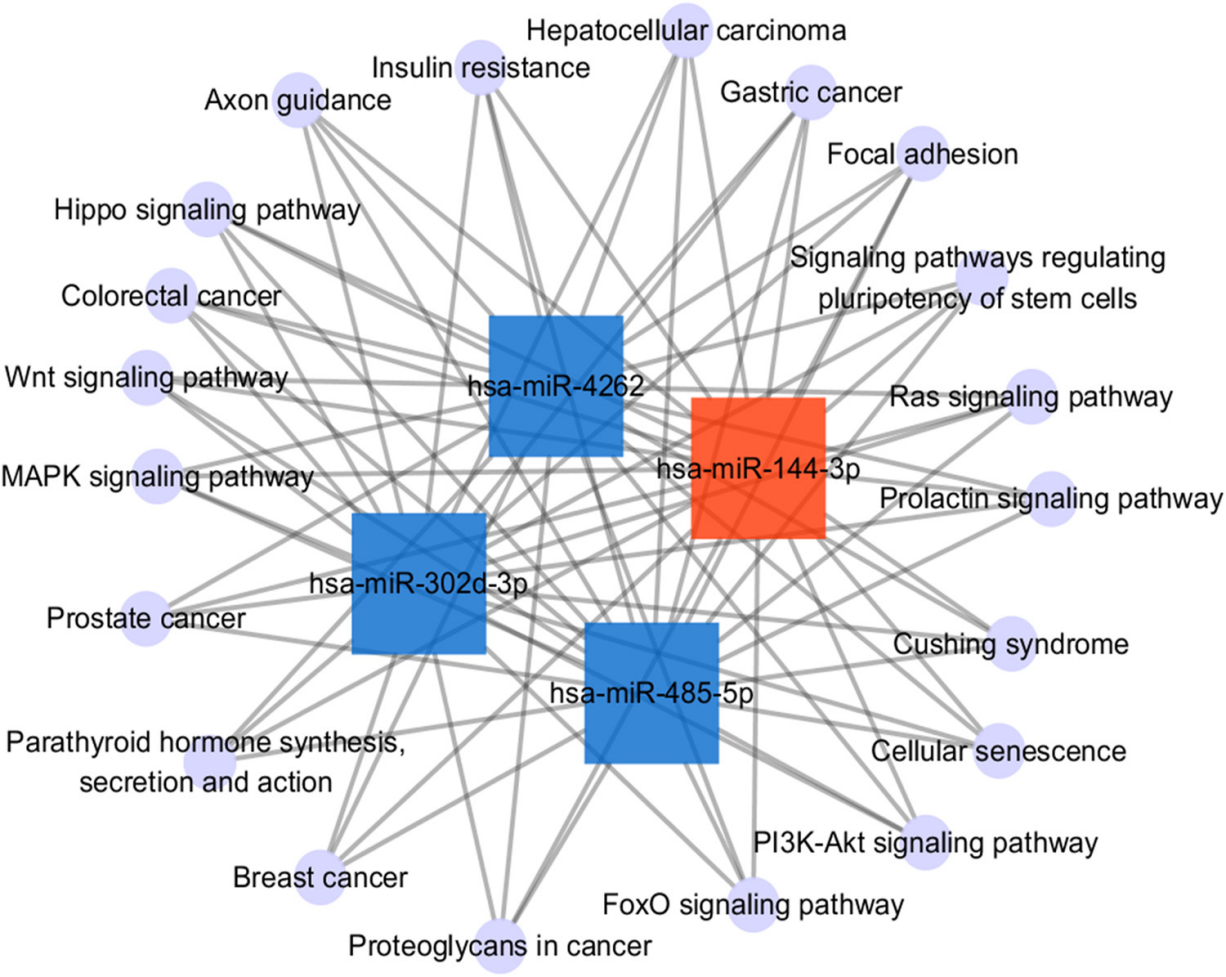
Interaction between the top 20 KEGG pathways and the differentially expressed miRNAs. The upregulated miRNAs were represented as red square nodes, the downregulated miRNAs were represented as blue square nodes, and the KEGG pathways were represented as violet circular nodes.

KEGG pathway analysis of the differentially expressed miRNAs was used to identify target genes. Axon guidance was the most significant pathway according to *p*-values. In addition, the signaling pathways regulating pluripotency of stem cells, proteoglycans in cancer, focal adhesion, the microtubule associated protein kinase (MAPK) signaling pathway, and the phosphatidylinositol 3-kinase-protein kinase B (PI3K-Akt) signaling pathway were identified as differentially regulated pathways (Figure 4). To understand the potential pathway targets of the differentially expressed miRNAs better, the interaction between the top 20 significant KEGG pathways and the differentially expressed miRNAs was studied (Figure 5). The results showed a significant interaction between top 20 KEGG pathways and the differentially expressed miR-144-3p, miR-4262, miR-302d-3p, and miR-485-5p.

### 3.4 Validation by RT-qPCR

**Figure 6:**
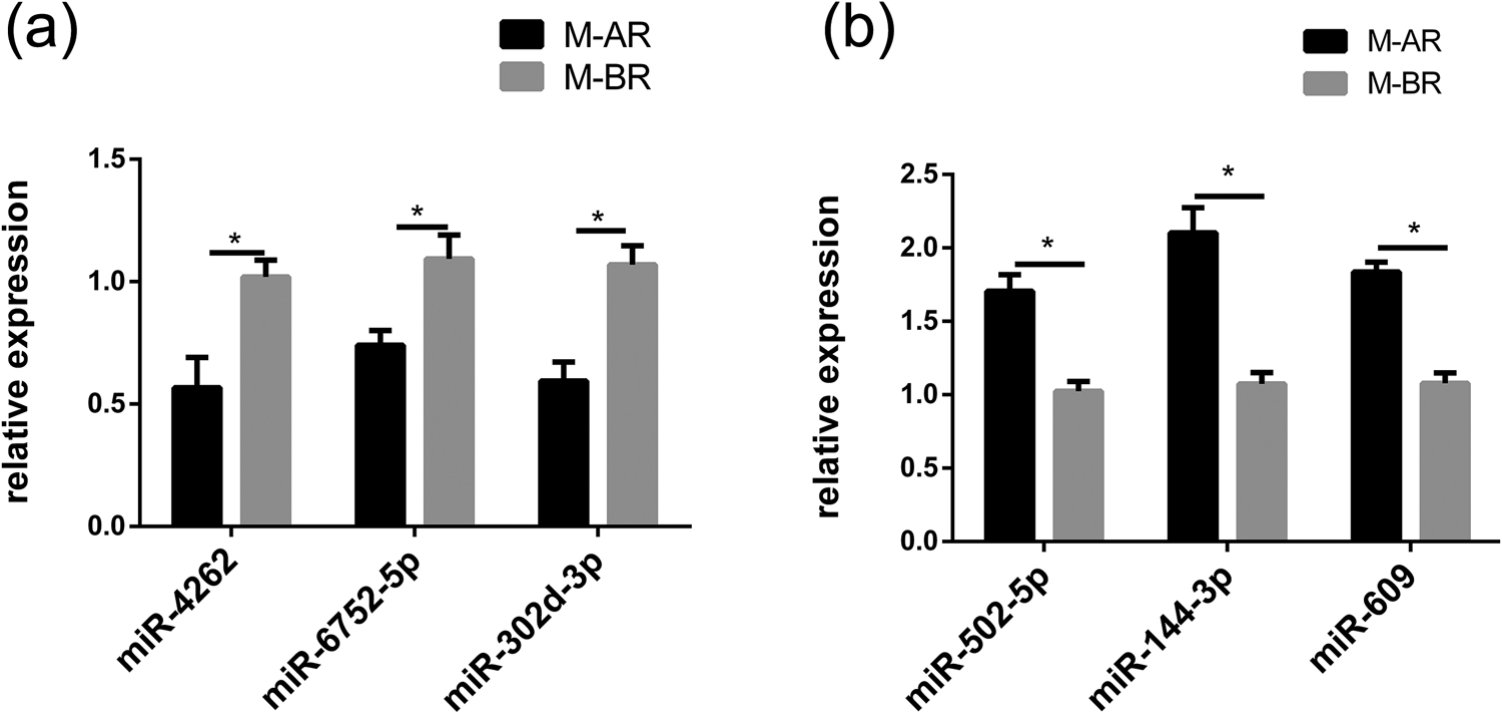
Validation of miRNA by RT-qPCR. (a) Downregulated miRNAs in the brain metastasis with radiotherapy group; (b) upregulated miRNAs in the brain metastasis with radiotherapy group. Compared with the control group, the expression of all miRNAs in the experimental group had significant changes. *p < 0.05.

To confirm and validate the expression of the significantly differentially expressed miRNAs identified in this study, we evaluated the expression of six miRNAs (Table 2) in the plasma exosomes of patients with brain metastasis using stem-loop RT-qPCR. The miRNAs were differentially expressed in the pre-and post-radiotherapy samples. The expression of three miRNAs (miR-4262, miR-6752-5p, and miR-302d-3p) decreased in the plasma exosomes of patients after radiotherapy. The expression of miR-502-5p, miR-144-3p, and miR-609 was higher in the plasma exosomes of pre-radiotherapy samples as compared to the post-radiotherapy samples (Figure 6).

**Table 2:** miRNA forward primer sequences

Accession number	Gene name	Target forward primer sequence (5′–3′)
MIMAT0016894	miR-4262	GACATTCAGACTACCTG
MIMAT0027404	miR-6752-5p	GGGGGGTGTGGAGCCAGGGGGC
MIMAT0000718	miR-302d-3p	TAAGTGCTTCCATGTTTGAGTGT
MIMAT0002873	miR-502-5p	ATCCTTGCTATCTGGGTGCTA
MIMAT0000436	miR-144-3p	TACAGTATAGATGATGTACT
MIMAT0003277	miR-609	AGGGTGTTTCTCTCATCTCT
NR_004394.1	U6	GCGCGTCGTGAAGCGTTC

## 4 Discussion

Exosomes are considered intercellular communicators that can promote the development of cancer, and they can be used potentially as biomarkers and in therapeutic methods. Exosomes play an important role in the tumor microenvironment interactions in primary and metastatic brain tumors [15]. Studies have reported that exosome integrin in tumors can be used to predict organ-specific metastasis [16]. In addition, cell migration-inducing and hyaluronan-binding (CEMIP) protein in tumor exosomes from brain metastasis, but not from lung or bone metastatic cells, may promote cancer cell colonization [17]. miRNAs can regulate gene expression by degrading mRNA or repressing mRNA translation. The intercellular exchange of miRNA through exosomes is a potential and effective method of intercellular communication that may have multiple functions, especially in tumor survival and metastasis. Exosomal miRNAs can induce phosphatase and tensin homolog (PTEN) loss in the microenvironment, thus promoting brain metastasis [18]. Currently, because of the limitations of our understanding of molecular mechanisms underlying brain metastasis, the development of effective treatments for brain metastasis is hindered. In this study, the changes in exosome miRNA expression resulting from radiotherapy performed for brain metastasis were defined for the first time.

This study identified 35 miRNAs that were differentially expressed in the five patients with brain metastasis before and after radiotherapy. Because of the limited sample size collected clinically, the study should be referred to as a “pilot” study. Besides, there are still many interesting results to be found, which could reflect the overall characteristics to a certain extent. *Homo sapiens* (hsa)-miR-4262 has been reported to participate in paclitaxel resistance in non-small cell lung cancer by regulating PTEN, and it promotes the proliferation and invasion of human breast cancer cells directly by targeting kruppel-like 6 (KLF6) and KLF15 [19,20]. hsa-miR-4262 was also expressed in five human melanoma cell lines, and its expression pattern was opposite to KLF6 expression. Bioinformatics analysis and KLF6-3′ UTR luciferase reporter gene analysis indicated that KLF6 was the direct target gene of miR-4262. The miR-4262 can significantly reduce the expression of KLF6 protein and promote the proliferation of melanoma cells [21]. Moreover, an abnormal increase in miR-4262 promotes cell proliferation and migration by targeting large tumor suppressor 1 in gliomas [22,23]. The expression of miR-302d-3p is increased by a low-Se environment in the Caco-2 cell line. Dysregulation of the cell cycle and of the stress response pathways caused by low Se may influence key genes involved in carcinogenesis. The effect of low Se on biological pathways may be partly because of the action of Se-sensitive miR-302d-3p [24]. It has been found that miR-4262 can be sponged by circAGFG1, and it can regulate the YY1/CTNNB1 axis to drive colorectal cancer metastasis [25]. In this study, the expression of miR-4262 and miR-302d-3p decreased in the pre-radiotherapy samples as compared to the pre-and post-radiotherapy samples, indicating that radiotherapy may inhibit the proliferation of cancer cells by reducing the expression of miR-4262 and miR-302d-3p in exosomes of patients with brain metastasis. Furthermore, hsa-miR-502-5p and hsa-miR-144-3p were upregulated after radiotherapy. The expression of miR-502 is regulated by a negative feedback action of p53, and the expression of miR-502-5p was found to be downregulated in colon cancer patients as compared to that in matched normal controls. Moreover, miR-502 inhibits the growth of colon cancer in mice. Studies have shown that miR-502 regulates autophagy by inhibiting RAB1B, which is the key mediator of autophagy, and miR-502-5p inhibits autophagy, growth, and cell cycle progression in colon cancer cells [26]. It has been reported that miR-144-3p plays different roles in various diseases and promotes tumor growth and metastasis of papillary thyroid carcinoma by targeting the paired box gene 8 [27]; However, it serves as a tumor suppressor in renal cell carcinoma and inhibits its invasion and metastasis by targeting MAP3K8, also a tumor suppressor, by targeting FZD7. Furthermore, these outcomes can be used to predict the prognosis of human glioblastoma [28,29]. These results suggest that miR-502 and miR-144-3p may be potential tumor suppressors and may have some function in the role of radiotherapy in brain metastasis treatment.

GO and KEGG pathway enrichment analysis showed the significantly enriched functions and pathways targeted by the differentially expressed miRNAs. In this study, the GO terms of regulation of cellular process, cellular macromolecule metabolic process, regulatory region nucleic acid binding, and transcription regulator activity were all enriched by the significantly differentially expressed miRNA-targeted genes. miRNAs can regulate mRNAs in different biological pathways, and miRNA expression may play a role in the modulation of anticancer effects via regulation of different cellular processes [30]. Metabolic changes are a common feature of invasive cancer cells. Cancer cells reprogram metabolic pathways to generate energy and macromolecules required for cell growth [31]. Therefore, cellular macromolecule metabolic processes are of great significance to cancer cells. Regulatory region nucleic acid binding modulates the tumor microenvironment in colorectal cancer tumorigenesis [32]. Transcription regulator activity can regulate the proliferation or apoptosis of cancer cells by affecting cell proliferation-related genes and directly regulating the cell cycle [33,34]. Using the KEGG pathway, axon guidance promoted perineural invasion and metastasis of orthotopic pancreatic tumors in mice and regulated the tumor microenvironment [35,36]. Furthermore, the signaling pathways regulating the pluripotency of stem cells and the MAPK and PI3K-Akt signaling pathways are closely related to the efficient differentiation of endothelial cells [37]. Proteoglycan regulates focal adhesion dynamics and cell motility [38]. These results suggest that the miRNAs identified in plasma exosomes affected by radiotherapy may be involved in the regulation of genes related to neurodevelopment, proliferation, or apoptosis of tumor cells in brain metastasis.

## 5 Conclusion

This study reported the first description of miRNAs in plasma exosomes from patients with brain metastasis who had undergone radiotherapy. Although the sample size of the study was small, it provides a possible role of plasma exosomes as a potential therapeutic target for brain metastasis. Besides, it is time to go a step further to explore the discovery and application of plasma exosomes as noninvasive biomarkers in the treatment of brain metastasis with large sample size.
